# Dosimetric analysis of 17 cardiac Sub-structures, Toxicity, and survival in ultra central lung tumor patients treated with SBRT

**DOI:** 10.1016/j.ctro.2023.100675

**Published:** 2023-09-13

**Authors:** Maiwand Ahmadsei, Kai Thaler, Elena Gasser, Bertrand Pouymayou, Riccardo Dal Bello, Sebastian M. Christ, Jonas Willmann, Boldizsar Kovacs, Panagiotis Balermpas, Stephanie Tanadini-Lang, Ardan M. Saguner, Michael Mayinger, Nicolaus Andratschke, Matthias Guckenberger

**Affiliations:** a Department of Radiation Oncology, University Hospital Zurich, University of Zurich, Rämistrasse 100, 8091 Zurich, Switzerland; b Department of Cardiology, University Heart Center, University Hospital Zurich, University of Zurich, Zurich, Switzerland; c Center for Translational and Experimental Cardiology (CTEC), Department of Cardiology, Zurich University Hospital, University of Zurich, 8952 Schlieren, Switzerland

**Keywords:** SBRT, Cardiac toxicity, Ultra-central lung tumors, Cardiac sub-structures, Stereotactic body radiotherapy

## Abstract

• Data on cardiac toxicity after SBRT for ultra-central lung tumors remains limited.
• We analyzed the dose to 18 cardiac sub-structures and cardiovascular toxicity.
• A SBRT regimen of 45 Gy in 8–10 fractions yields good local control and low toxicity.
• The highest cardiac doses were observed in the pulmonary artery and left atrium.
• Higher doses to the base of the heart seem to be associated with non-cancer deaths.

## 1 Introduction

With an estimated 1.8 million deaths worldwide in 2020, lung cancer has the highest cancer-related mortality [1]. Stereotactic body radiotherapy (SBRT) is the treatment of choice for patients with inoperable early stage non-small cell lung cancer (NSCLC), operable stage I-II NSCLC - if surgery is refused by the patient - and oligometastatic (OMD) pulmonary disease [2], [3], [4], [5], [6], [7]. In the case of ultra-central lung tumors (UCLT), defined as tumor overlapping with the proximal bronchial tree, trachea or esophagus, previous studies reported favorable efficacy at the cost of an increased risk of grade ≥ 3 toxicity and treatment-related mortality [8], [9], [10]. As survival continues to improve with more effective systemic therapy, there is a growing concern about long-term radiotherapy (RT)-related toxicity [11], [12].

Recent studies demonstrated that thoracic RT may be associated with an increased risk of cardiotoxicity and non-cancer deaths [13], [14], [15], [16], [17]. The landmark clinical trial *RTOG 0617*, which compared standard thoracic RT (60 Gy/30 fractions) to a higher dose (74 Gy/37 fractions), identified V30 and V5 of the heart as prognostic factors for survival [18]. A post-hoc analysis demonstrated that the dose delivered to the base of the heart is an independent prognostic factor for all-cause mortality [19]. In a systematic review by *Tohidinezhad et al.*, which analyzed 28 prediction models for radiotherapy-induced cardiac toxicity in patients with NSCLC, the authors identified the mean heart dose (MHD) and a history of cardiovascular diseases as factors significantly associated with cardiac toxicity after (chemo-)radiotherapy [20].

However, data on cardiac toxicity is mostly based on patients treated with conventionally fractionated radiotherapy, where usually large volumes of heart are exposed to rather low radiation doses [21], [22]. Data on cardiac toxicity following small-volume high-dose per fraction SBRT remains scarce, especially for UCLT patients, where high dose exposure of small heart subvolumes is expected [23], [24], [25]. In the absence of uniform delineation of cardiac sub-structures in existing literature, recent studies have demonstrated the potential of machine learning methods to automatically and accurately delineate cardiac substructures, thereby providing increased standardization, improved comparability and wider clinical use [26].

Therefore, this study aims to report the doses to the different substructures of the heart using a novel open source deep learning (DL)-based model to automatically segment the heart and 17 cardiac sub-structures and to report the cardiac toxicity in regard to the dose to cardiac sub-structures associated with SBRT treatment in patients with UCLT.

## 2 Material and methods

### 2.1 Patient selection

**Fig. 1:**
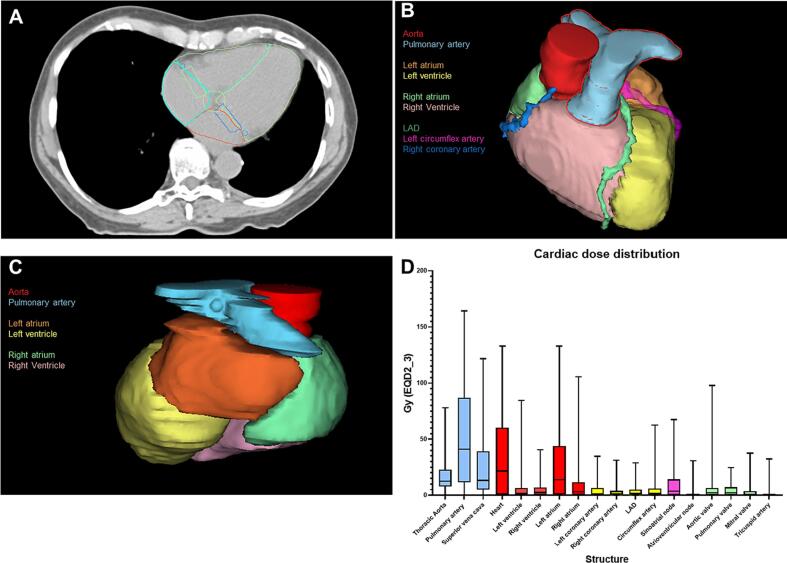
**(A-D):** (A) auto-segmentation of the heart and 17 heart substructures on planning CT, (B) 3D visualization of the auto-segmented heart substructures (anterior), (C) posterior view of 3D visualization of the auto-segmented heart substructures, (D) cardiac dose distribution (D0.1 cc in EQD2_3), showing large vessels highlighted (blue), cardiac chambers (red), coronary arteries (yellow), pacemaker regions (purple) and heart valves (green).

All UCLT patients treated with SBRT between 2014 and 2021 were included in this study. Ultra-central location was defined as the PTV overlapping or abutting the PBT, trachea or esophagus (Fig. 1**A**). The patients presented either with primary inoperable NSCLC, loco-regionally recurrent NSCLC or (oligo-) metastases. This study was approved by the Swiss Cantonal Ethics Committee before study initiation (BASEC# 2018–01794).

### 2.2 Treatment planning and delivery

All patients were treated according to our institutional radiotherapy (RT) protocol. Three-dimensional (3D) and four-dimensional (4D) computer tomography (CT) simulation was conducted to assess breathing motion using a Siemens SOMATOM Definition AS Open (Siemens AG, Germany). Thoracic organs at risk (OAR) were delineated according to RTOG 0236/ROSEL[27], dose volume constraints were applied according to the institutional protocol. The GTV was delineated by registering FDG-PET/CT and the planning CT using the lung window in ARIA® (Varian Medical Systems, Palo Alto, CA). The GTV was contoured on the end-expiration phase and the end-inspiration phase, the internal target volume (ITV) was defined as the fusion of these two contours. The ITV-to-PTV margin was 5 mm. The RTOG’s conformity index (CI) was defined as the 100% isodose volume divided by the PTV volume. A detailed description of prescribed doses is shown in Table 2. The treatment was delivered using a TrueBeamTM linear accelerator with daily cone-beam CT based image-guided set-up. All treatments were performed with Volumetric modulated arc therapy (VMAT, or RapidArc in Varian terminology). Multiple arcs were used, either with two full gantry rotations or multiple partial rotations summing up to a total of 720° covered by the VMAT arcs. Jaw tracking was activated in all cases.

**Table 1:** Patient and treatment characteristics.

**Parameter**	**Results (%)**
**Total number of patients** **Age at primary diagnosis in years, median (range)**	60 (1 0 0) 67.7 (33–83)
**Male gender, n (%)** **Median follow-up time in years (range)** **ECOG, median (range)**	45 (75.0) 2.2 (0.6–9.3) 1 (0–2)
**Primary tumor histology and disease stage, n (%)**	
**NSCLC** Primary, non-metastatic NSCLC Adenocarcinoma Squamous-cell carcinoma Loco-regionally recurrent NSCLC Adenocarcinoma Squamous-cell carcinoma Large-cell carcinoma **Oligometastatic disease** NSCLC Colorectal adenocarcinoma Head-and-Neck cancer Melanoma Sarcoma Other^1^ **Polymetastatic disease** NSCLC	27 (45.0) 12 (20.0) 5 (8.3) 7 (11.7) 15 (25.0) 10 (16.7) 4 (6.7) 1 (1.7) 30 (50.0) 10 (17.5) 4 (6.7) 4 (6.7) 3 (5.0) 3 (5.0) 6 (10) 3 (5.0) 3 (5.0)
**COPD** Gold 1 Gold 2 Gold 3 Gold 4	21 (35.0) 3 (5.0) 8 (13.3) 7 (11.7) 3 (5.0)
**OMD status (all patients)** De-novo Repeat Induced	30 (50.0) 8 (13.3) 13 (21.7) 9 (15.0)
**Alive at time of analysis, n (%)** **Radiotherapy of primary tumor, n (%)** **Radiotherapy of pulmonary metastasis, n (%)** **Prior treatment** Surgery Radiotherapy Type-I re-irradiation Chemotherapy Immunotherapy Targeted therapy Cardiotoxic systemic therapy* Systemic therapy < 6 months before index radiotherapy	21 (35.0) 27 (45.0) 33 (55.0) 47 (78.3) 29 (48.3) 22 (36.7) 13 (21.7) 25 (41.7) 14 (23.3) 5 (8.3) 17 (28.3) 19 (31.7)
**Cardiovascular comorbidities at baseline, n (%)** Coronary artery disease Ischemic heart disease Prior myocardial infarction Non-coronary atherosclerosisNon-ischemic heart disease (congestive heart failure)Peripheral Arterial Disease (PAD)^2^ Pulmonary hypertension Valvulopathy Prior stroke / TIA Atrial fibrillation Other arrhythmias^3^ Hypertension Dyslipidemia Diabetes mellitus Smoking history	44 (73.3) 13 (21.7) 1 (1.7) 3 (5.0) 10 (16.7) 8 (13.3) 11 (18.3) 1 (1.7) 9 (15.0) 4 (6.7) 9 (15.0) 10 (16.7) 30 (50.0) 11 (18.3) 10 (16.7) 48 (80.0)
**Treatment volume characteristics** Longest tumor diameter in cm, (range) <3 cm 3–7 cm >7 cm PTV location Overlap with PBT Overlap with trachea Overlap with heart Overlap with esophagus Overlap with Aorta Overlap with pulmonary artery PTV to heart distance in cm, median (range) Distance < 1 cm, n (%)	16 (26.7) 40 (66.7) 4 (6.7) 60 (100.0) 14 (23.3) 15 (25.0) 11 (18.3) 16 (26.7) 44 (73.3) 1.2 (0–6.3) 24 (40.0)
**Treatment characteristics** Single dose in Gy, median (range) Number of fractions, median (range) Total dose in Gray, median (range) EQD2_10 dose in Gray, median (range) Prescription Isodose, mode (%, range) V100% of PTV in %, median (range) D0.1 cc of PTV in EQD2_10 in Gy, median (range) GTV size in cm^3^, median (range) PTV size in cm^3^, median (range) Most frequent fractionation schemesEight fractions (8fx) 8 × 6 Gy@65% 8 × 5 Gy@65% Ten fractions (10fx) 10 × 5 Gy@80% 10 × 4.5 Gy@80%	5 (3–7.5) 8 (5–12) 45 (30–60) 55.2 (33.0 – 88.0) 65 (65–80) 96.0 (9.5–99.4) 86.5 (43.1–120.6) 12.5 (0.6–114.9) 30.0 (6.0–199.0) 30 (50.0) 13 (21.7) 13 (21.7) 25 (41.7) 9 (15.0) 7 (11.7)

1 Includes small-cell lung cancer (SCLC), prostate cancer, mesothelioma, pancreatic cancer and urothelial cancer.

2 Includes ectasia and aneurysm.

3 Includes AV-block, left/right bundle branch block and bifascicular block. *Cardiotoxic agents included: Taxanes (Docetaxel, Paclitaxel), Antimetoblites (5-Fluoruracil), MEK inhibitors (Dabrafenib), Anthracyclines (Epirubicin), EGFR-TKI (Osimertinib) and other TKIs (Pralsetinib, Dabrafenib, Crizotinib).

**Table 2:** Oncological outcome and toxicity after index radiotherapy.

**Parameter**	**All patients (n = 60)**
**Median survival from time of radiotherapy years, (range)**2-year survival (%)	2.9 (0.6–9.3) 65.9
**Median local control in years, (range)**1-year local control rate (%)2-year local control rate (%)	*Not reached* 84.4 76.8
**Median distant control in years, (range)**1-year distant control rate (%)2-year distant control rate (%)	1.4 (0.2–5.7) 58.0 45.0
**Median PFS in years, (range)**	0.9 (0.2–5.7)
**All deaths** **Non-cancer deaths, n (%)**	39 (65.0) 6 (10.0)
**Treatment after index radiotherapy** Surgery Radiotherapy Chemotherapy Immunotherapy Targeted therapy	36 (60.0) 5 (8.3) 22 (36.7) 20 (33.3) 15 (25.0) 7 (11.7)
**Systemic therapy < 6 months after index radiotherapy**	24 (40.0)
**Treatment-related toxicity** *Type of toxicity* Radiation pneumonitis Grade 3 Grade 4 Grade 5 Bronchial stenosis Grade 3–5 Bronchopulmonary hemorrhage Grade 3–5 Fistula formation Grade 3–5 Esophagitis Grade 3–5	1 (1.7) 1 (1.7) 0 0 0 0 0

### 2.3 Data collection and outcome measurement

All patients of this study were identified via the institutional SBRT database. Patient information, baseline cardiovascular risk profile and treatment characteristics were extracted from the institutional hospital information system KISIM^TM^ and our treatment planning system External Beam Planning® (Varian, A Siemens Healthineers Company). Toxicity assessment after treatment was conducted according to Common Terminology Criteria for Adverse Events (CTCAE) Version 5. All grade ≥ 3 toxicities were documented in detail with date of occurrence and therapeutic management. The present study included all previously known cardiovascular disease and cardiovascular risk factors known, such as smoking history, hypertension, diabetes mellitus and dyslipidemia, if this information was available based on previous medical reports. Occurrence of cardiovascular events (CVE) and non-cancer death was documented during follow-up, which was conducted six weeks after completion of treatment and afterwards every three months. Freedom from local progression (FFLP), progression-free survival (PFS) and freedom from distant progression (FFDP) were assessed using regular follow-up Fluorodeoxyglucose (18F)-Positron emission tomography–computed tomography (FDG-PET/CT), prostate-specific membrane antigen (PSMA)-PET/CT or CT, which were conducted every three months during follow-up.

### 2.4 Automated segmentation of the heart and 17 cardiac sub-structures

To increase spatial resolution of dose delivered to the heart and standardize delineation, we conducted fully-automated hybrid cardiac substructure segmentation of the heart and 17 cardiac sub-segments using the novel and open-source model by *Finnegan et al.* **(**Fig. 1**B-C)** [26]. Structures were defined per the atlas described by *Feng et al.* [16]. The segmentation model entails a hybrid algorithm using a deep learning model (nnU-Net)[28] to segment the whole heart, followed by a multi-atlas based mapping of the cardiac substructure and in the final step geometric modeling of smaller cardiac structures. Each structure (whole heart, cardiac chambers, great vessels, heart valves, coronary arteries, and conduction nodes) was manually reviewed by a senior radiation oncology resident and edited if necessary. Eclipse (Varian Medical Systems, Palo Alto, CA, USA) was used for the generation and evaluation of radiation treatment plans.

### 2.5 Statistical analysis

Overall survival (OS) was measured from the time of completion of treatment until death or last follow-up. PFS was measured from the time point of completion of SBRT until locoregional relapse, distant disease progression, death, or the last follow-up. FFLP and FFDP were measured from the time of treatment completion until local/distant disease progression or last follow-up. OS, FFLP and PFS curves were estimated by using Kaplan-Meier method and compared by log-rank test. Furthermore, univariate and multivariate analysis were performed using the Cox proportional hazard model. Dose volume histograms (DVHs) were extracted from the treatment planning system for dosimetric analysis. EQD2 sum plans were calculated for all courses of thoracic radiotherapy using the software solutions Eclipse and R-Studio statistical software using the following formula: EQD2 = D*(D/n_fx + alpha/beta)/(2 + alpha/beta). All statistical analysis were conducted in in R-Studio statistical software (Version 2022.12.0 + 353, R-packages “survival” and “dvhmetrics”) and MedCalc statistical software (Version 20.305, MedCalc Software Ltd). Statistical significance was set at p < 0.05. Correction for multiple testing was conducted using Benjamini-Hochberg procedure.

## 3 Results

### 3.1 Patient cohort and cardiovascular baseline characteristics

A total of 60 UCLT patients were included in this study. The median age was 67.7 (range: 33–83) years. The most common primary tumor was primary non-metastatic and loco-regionally recurrent NSCLC (n = 27, 45%), followed by oligometastatic NSCLC (n = 10, 17.5%). A total of 33 (55%) irradiated targets were pulmonary metastases and 27 targets (45%) were primary lung tumors. Seventeen patients (28.3%) had received cardiotoxic systemic therapy (induction, concurrent or adjuvant). A total of 44 patients (73.3%) presented with cardiovascular comorbidities, most commonly in the form of hypertension in 30 cases (50%) and dyslipidemia in 11 cases (18.3%). A detailed description of patient characteristics is shown in Table 1.

### 3.2 Treatment parameters

The median PTV size was 30.0 (range: 6.0–199.0) cm^3^, the median distance from PTV to the heart was 1.2 (range: 0–6.3) cm, in 24 cases (40.0%) the PTV-to-heart distance was < 1 cm. In 14 cases (23.3%) the PTV overlapped with the heart. The most commonly used fractionations were 8 × 6 Gy@65% (22.8%), 8 × 5 Gy@65% (22.8%), 10 × 5 Gy@80% (14.0%) and 10 × 4.5 Gy @80% (10.5%). A detailed description of the treatment characteristics is shown in Table 1.

### 3.3 Detailed dosimetric analysis of the heart and 17 cardiac sub-regions

The MHD (stated in EQD2_3 as for all structures) was 0.8 (range: 0.04–9.9) Gy, while the median D_0.1cc_ of the heart was 21.6 (range: 0.01–133.0) Gy (Fig. 1**D**). Within the heart chambers, the left atrium showed the highest median D_0.1cc_ of 13.9 (range: 0.11–133.0) Gy, while the median D_0.1cc_ of the left ventricle was 1.7 (0.01–88.0) Gy. The median D01.cc to the right atrium and right ventricle were 3.1 (range: 0.1–105.6) Gy and 2.5 (0.06–40.6) Gy, respectively. Furthermore, the median D_0.1cc_ to the left coronary artery, right coronary artery and left circumflex artery were 1.1 (range: 0.08–34.8) Gy, 1.4 (range: 0.01–32.3) Gy and 1.9 (range: 0.08–62.5) Gy, respectively. The highest dose observed (median D_0.1cc_) to any cardiac sub-structure was observed for the pulmonary artery with 41.2 (range: 0.06–164.2) Gy. The laterality of the tumor (right side: 27/60, left side: 21/60 and central/tracheal: 12/60) had no impact on the dose delivered to the heart (p = 0.45). A detailed overview of dose distribution to the heart and 17 cardiac sub-structures are shown in Table 3 and Fig. 1**D**.

**Table 3:** Cardiovascular outcome after index radiotherapy and dosimetric analysis of the heart and 17 heart sub-segments of all patients in EQD2_3.

**Symptoms assessed at last clinical visit** **Dyspnea NYHA class, n (%)** I II III IV **Orthopnea, n (%)** **Lower extremity edema, n (%)** **Palpitation, n (%)** **Syncope, n (%)**	5 (8.3) 9 (15.0) 6 (10.0) 8 (13.3) 3 (5.0) 4 (6.7) 0 (0.0) 1 (1.7)	
**Cardiovascular event after RT, n (%)** Any new onset of cardiovascular event CVD-related death, n (%) Non-fatal myocardial infarct, n (%) Non-fatal stroke, n (%) Coronary artery disease, n (%) Congestive heart failure, n (%) Valvulopathy, n (%) Pericardial disease, n (%) Atrial fibrillation, n (%) Other arrhythmic disease, n (%) Pulmonary embolism, n (%) Peripheral thrombo-embolic event, n (%)	12 (20.0) 1 (1.7) 0 (0.0) 1 (1.7) 1 (1.7) 2 (3.3) 6 (10.0) 1 (1.7) 5 (8.3) 0 (0.0) 3 (5.0) 0 (0.0)	**Median time between onset and RT in years** 1.8 (0.2–6.1) 2.3 - 0.2 2.1 2.5 1.8 1.2 0.4 - 1.1 -
**Dosimetric analysis**		
Structure	Parameter	All patients in Gy (EQD2_3), median (range)
Heart	D_1cc_ D_0.5cc_ D_0.1cc_ D_mean_ D_max (pointdose)_	16.6 (0.01–107.6) 18.5 (0.01–122.2) 21.6 (0.01–133.0) 0.8 (0.04–9.9) 25.1 (0.12–138.2)
**Heart Chambers**		
Left ventricle	D_0.1cc_ D_mean_	1.7 (0.01–88.0) 0.20 (0.02–7.1)
Right ventricle	D_0.1cc_ D_mean_	2.5 (0.06–40.6) 0.16 (0.03–5.5)
Left atrium	D_0.1cc_ D_mean_	13.9 (0.11–133.0) 1.0 (0.04–21.7)
Right atrium	D_0.1cc_ D_mean_	3.1 (0.10–105.6) 0.3 (0.03–17.7)
**Coronary Arteries**		
Left coronary artery	D_0.1cc_ D_mean_	1.1 (0.08–34.8) 0.7 (0.02–15.0)
Right coronary artery	D_0.1cc_ D_mean_	1.4 (0.01–31.3) 0.4 (0.04–11.6)
LAD	D_0.1cc_ D_mean_	1.6 (0.02–28.8) 0.4 (0.03–5.0)
Circumflex artery	D_0.1cc_ D_mean_	1.9 (0.08–62.5) 0.8 (0.04–30.0)
**Large Vessels**		
Ascending aorta	D_0.1cc_ D_mean_	12.7 (0.06–78.0) 3.0 (0.02–34.1)
Pulmonary artery	D_0.1cc_ D_mean_	41.2 (0.06–164.2) 5.1 (0.01–23.0)
Superior vena cava	D_0.1cc_ D_mean_	13.2 (0.18–121.7) 4.1 (0.01–68.0)
**Conduction nodes**		
Atrioventricular node	D_0.1cc_ D_mean_	0.3 (0.04–30.7) 0.20 (0.03–16.1)
Sinoatrial node	D_0.1cc_ D_mean_	3.7 (0.06–67.5) 2.6 (0.02–49.2)
**Heart Valves**		
Aortic valve	D_0.1cc_ D_mean_	2.17 (0.07–97.9) 0.4 (0.02–23.8)
Pulmonary valve	D_0.1cc_ D_mean_	2.1 (0.1–24.6) 1.4 (0.03–16.0)
Mitral valve	D_0.1cc_ D_mean_	0.6 (0.05–37.5) 0.3 (0.03–15.4)
Tricuspid valve	D_0.1cc_ D_mean_	0.2 (0.04–32.4) 0.2 (0.03–8.7)

### 3.4 Control rates, overall survival and progression-free survival

**Fig. 2:**
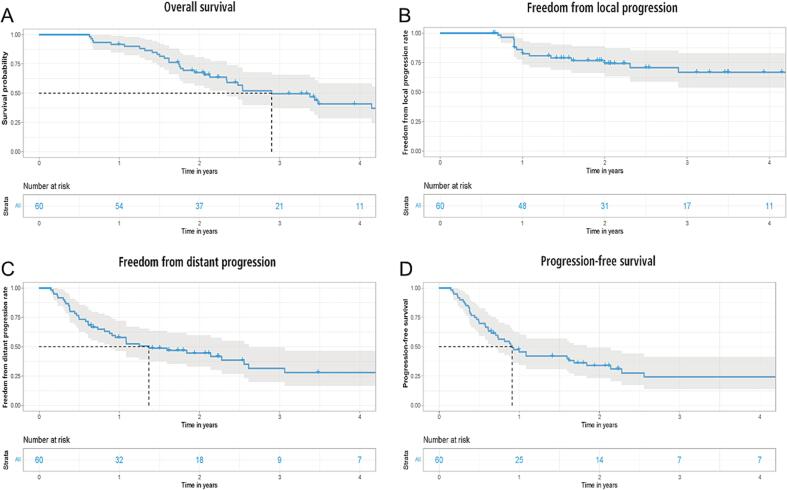
**(A-D):** (A) overall survival for all patients, (B) freedom from local progression for all patients, (C) freedom from distant progression for all patients and (D) progression-free survival for all patients.

After a median follow-up time of 2.2 (range: 0.6–9.3) years, 39 patients (65%) were dead, non-cancer deaths accounted for six cases (10%), one of them being fatal heart failure, one fatal sepsis, two fatal COPD exacerbation, one fatal septic atrial fibrillation and one fatal aspiration. The median OS was 2.9 (0.6–9.3) years. The two-year survival was 65.9%, while the one-year and two-year FFLP rates were 84.4% and 76.8%, respectively (Fig. 2**A-C**), the median PFS was 0.9 (range: 0.2–5.7) years (Fig. 2**D**). Chemotherapy, immunotherapy and targeted therapy were administered after index RT in 20 (33.3%), 15 (25%) and 7 (11.7%) cases, respectively. A summary of treatment received after index RT is shown in Table 2.

In the univariate Cox regression analysis, only tumor size (HR: 1.4, p = 0.0049) was associated with worse OS. In multivariate Cox regression analysis tumor size (HR: 1.37, p = 0.00457), age (HR: 1.04, p = 0.047) and polymetastatic disease (HR: 6.17, p = 0.01178) were significantly associated with shorter OS. The heart dose and dose delivered to any cardiac sub-structure were not predictive of OS, as shown in Table 4.

**Table 4:** Univariate and multivariate Cox regression analysis of clinical and dosimetric parameters associated with overall survival and non-cancer death.

**Endpoint**	**Overall survival HR (95% CI)**				**Non-cancer death HR (95% CI)**	
*Structure*	UVA - HR (95% CI)	P value	MVA - HR (95% CI)	P value	UVA - HR (95% CI)	P value
Age	1.01 (1.0–1.1)	0.27	**1.04 (1.0**–**1.1)**	**0.04756**	1.0 (0.95–1.1)	0.5
KPS	1.04 (1.0–1.1)	0.47	**–**	**–**	1.0 (0.98–1.1)	0.5
Sex Female Male	*Reference* 1.2	- 0.82	– -	– -	- 1.8 (0.21–15)	- 0.6
Tumor size	**1.4 (1.2**–**1.7)**	**0.005**	**1.37 (1.1**–**1.7)**	**0.00457**	1.5 (1–2.3)	0.053
PTV to heart distance	1.04 (0.84–1.3)	0.82	1.1 (0.84–1.3)	0.653	0.75 (0.4–1.5)	0.398
Primary diagnosis NSCLC (primary and non-metastatic) OMD Polymetastatic disease	*Reference* 0.5 (0.2––1.1) 5.2 (1.4–19.0)	0.31 0.16	0.71 **6.17 (1.5**–**25.4)**	0.41 **0.01178**	0.5 (0.09–3.2) 10.2 (1.8–128.0)	0.49 0.07
Cardiotoxic systemic therapy	1.2 (0.5–2.8)	0.82	–	–	0.69 (0.08–6.1)	0.74
Cardiovascular risk factor at baseline	1.3 (0.6–3.0)	0.82	–	–	1.2 (0.7–2-8)	0.54
Heart (D1cc) Heart (D0.5 cc) Heart (D0.1 cc) Heart (DMAX) Heart (DMEAN)	1.0 (0.99–1) 1.0 (0.99–1) 1.0 (0.99–1) 1.0 (0.99–1) 1.1 (0.94–1.3)	0.82 0.82 0.82 0.82 0.62	- - - - -	- - - - -	1.0 (0.98–1) 1.0 (0.98–1) 1.0 (0.98–1) 1.0 (0.98–1) 1.1 (0.79–1.6)	0.83 0.82 0.73 0.68 0.54
Left atrium (D0.1 cc)	1.0 (1.0–1.0)	0.62	–	–	1.0 (0.98–1)	0.7
Right atrium (D0.1 cc)	0.99 (0.98–1.0)	0.81	–	–	1.0 (0.97–1)	0.91
Right atrium (D45%)	1.0 (0.9–1.1)	0.97	–	–	0.87 (0.5–1.5)	0.62
Left ventricle (D0.1 cc)	1.0 (1.0–1.0)	0.31	–	–	0.99 (0.9–1.1)	0.81
Right ventricle (D0.1 cc)	1.0 (0.97–1.0)	0.82	–	–	1.0 (0.94–1.1)	0.63
Right ventricle (D45%)	1.1 (0.79–1.6)	0.82	–	–	1.1 (0.46–2.5)	0.88
Left coronary artery (D0.1 cc)	1.1 (1.0–1.1)	0.27	1.04 (0.98–1.1)	0.16516	1.0 (0.92–1.2)	0.49
Left anterior descending artery (D0.1 cc)	1.0 (0.98–1.1)	0.49	–	–	1.1 (0.92–1.2)	0.44
Left circumflex artery (D0.1 cc)	1.01 (1.0–1.1)	0.16	1.02 (1.0–1.1)	0.178	1 (0.92–1.1)	0.98
Right coronary artery (D0.1 cc)	1.0 (0.95–1.1)	0.82	–	–	1 (0.88–1.2)	0.8
Aortic valve (D0.1 cc)	0.99 (0.96–1)	0.82	–	–	0.99 (0.93–1.1)	0.82
Pulmonary valve (D0.1 cc)	1.0 (0.99–1.1)	0.31	–	–	1.1 (0.95–1.2)	0.31
Mitral valve (D0.1 cc)	1.0 (1–1.1)	0.31	–	–	0.99 (0.87–1.1)	0.84
Tricuspid valve (D0.1 cc)	1.0 (0.99–1)	0.86	–	–	0.9 (0.64–1.3)	0.55
Sinoatrial node (D0.1 cc)	1.0 (0.98–1)	0.97	–	–	1 (0.99–1.1)	0.19
Atrioventricular node (D0.1 cc)	0.99 (0.96–1)	0.82	–	–	0.98 (0.9–1.1)	0.7
Superior vena cava (D0.1 cc)	1.0 (0.98–1)	0.82	–	–	1 (0.97–1)	0.99
Superior vena cava (DMEAN)	1.0 (0.97–1.0)	0.82	–	–	**1.01 (1**–**1.1)**	**0.04**
Pulmonary artery (D0.1 cc)	1.0 (0.99–1.0)	0.31	–	–	1 (1–1)	0.091
Pulmonary artery (DMEAN)	1.1 (1.0–1.1)	0.26	–	–	**1.20 (1.1**–**1.4)**	**0.0026**
Aorta (D0.1 cc)	1.0 (0.99–1)	0.33	–	–	1 (0.99–1.1)	0.15

Sex, primary diagnosis, cardiotoxic systemic therapy, cardiovascular risk factors were defined as categorical variables, while all other variables were defined as continuous. Correction for multiple testing was conducted using Benjamini-Hochberg procedure.

However concerning non-cancer death, in the univariate analysis the D_mean_ of the superior vena cava (HR: 1.01, CI: 1–1.1, p = 0.04) and pulmonary artery (HR: 1.2, CI: 1.1–1.4, p = 0.0026) were significantly associated with non-cancer death (Table 4). Due to the insufficient number of non-cancer death events for multivariate analysis, we have compared the median (D_mean_) dose to the pulmonary artery and superior vena cava between patients experiencing non-cancer death and all other patients. With a median Dmean of 11.3 (2.0–23.0) Gy vs. 4.6 (0.02–21.3) Gy for the pulmonary artery (p = 0.00072) and 10.7 (1.5–68.0) Gy vs. 4.1 (0.01–21.3) Gy to the superior vena cava (p = 0.021) patients experiencing non-cancer death showed significantly higher Dmean doses to the aforementioned structures.

### 3.5 General and cardiovascular RT-associated toxicity

Only two patients (3.3%) developed RT-associated pulmonary toxicity in the form of late grade 3 and grade 4 radiation pneumonitis (Table 2) during follow-up.

A total of 12 patients (20.0%) developed a CVE after index RT with a median of 1.8 (range: 0.2–6.1) years between disease onset and RT. Among the patients which developed new CVE, valvulopathy (n = 6, 10%) after a median onset time of 1.8 years and atrial fibrillation (n = 5, 8.3%) after 0.4 years, were the most common manifestations. A detailed overview of cardiovascular outcome after SBRT is shown in Table 3. Univariate Cox regression analysis could not detect any dosimetric or clinical variable associated with onset of new CVE (p=>0.05, *data not shown*).

## 4 Discussion

In this study, UCLT patients treated with SBRT showed a median OS of 2.9 (range:0.6–9.3) years, with a LC rate of 76.8% - in line with results reported in the literature. No severe ≥ grade 3 pulmonary toxicity classically associated with SBRT in UCLT, such as hemorrhage, fistula and stenosis was observed. While the majority of patients (n = 44; 73.3%) had any cardiovascular comorbidities at baseline, a total of 12 patients (20%) showed a CVE during follow-up. The median MHD dose of the present study was 0.8 Gy, in line with previously reported doses for the heart in early-stage lung cancer, yet the median D_0.1cc_ to the heart with 21.6 Gy vs. 13.1 Gy was higher than reported for central and peripheral tumor locations [29].

As reported for SBRT for central locations, the highest dose delivered to any cardiac sub-structure was observed for pulmonary arteries with a median D_0.1cc_ of 41.2 Gy [30]. While tumor size was significantly associated with decreased OS in univariate Cox regression analysis, cardiac sub-structure dosimetric parameters were not associated with decreased survival or occurrence of new cardiovascular disease. However, higher D_MEAN_ doses to the superior vena cava (HR: 1.25; p = 0.007) and pulmonary artery (HR: 1.06; p = 0.0205) were significantly associated with non-cancer death in univariate Cox regression analysis. This observation must be interpreted with caution due to the limited number of non-cancer deaths (n = 6), nevertheless all patients experiencing non-cancer death showed significantly higher doses delivered to the aforementioned cardiac sub-structures.

Results of our study, which could not establish a causal relationship due to the limited number of events, seem to be supported by findings in the literature, *Stam et al.*, who investigated the association of dose to the whole heart and cardiac sub-structures in 803 early stage NSCLC after SBRT, reported that higher doses to the left atrium and the superior vena cava were significantly associated with non-cancer death [17]. Additionally, the authors identified the upper region of the heart (atria and vessels) to be significantly associated with non-cancer death. In a recent study by *Farrugia et al.*, which analyzed the clinical consequences of dose delivered to the cardiac substructures during SBRT in early stage (NSCLC) central lung tumors, the authors reported that higher doses to the right atrium were associated with increased non-cancer deaths [30]. As the right atrium is anatomically in close proximity to the pulmonary artery and superior vena cava, the study by *Farrugia et al* [30], also reinforced the vulnerable role of the superior region of the heart, as the present study. Furthermore, *Wong et al*. demonstrated that higher biventricular doses were associated with poorer survival in central lung tumor patients after SBRT [31].

Several studies using conventionally fractionated RT seem to support our observation [21], *Ma et al.* reported that higher doses (V40-55) to the pulmonary artery were associated with impaired OS in patients with medically inoperable or unresectable NSCLC treated with definitive radiotherapy or chemoradiotherapy [32]. Yet, this finding must be treated with caution, as the pulmonary artery is the most commonly tumor-involved thoracic great vessel and tumor invasion of pulmonary artery is an independent factor associated with worse outcome [32], [33], [34], [35]. Therefore invasion of pulmonary artery, which deteriorates cardiac function, can be a confounding factor and a possible relationship between higher doses to pulmonary artery and tumor invasion of pulmonary artery can not be ruled out.

Other studies reported conflicting results regarding cardiac toxicity after SBRT for UCLT, making general conclusions problematic. This might be explained by the different timescale of manifestation of cardiac toxicity, radiation to coronary arteries result in atherosclerosis and its clinical manifestations are mainly important in long-term survivors, such as in breast cancer patients [29], [31], [36]. Against the background of the limited survival of a median of 2.9 years in the present study, it might explain why no association between radiation to coronary arteries and non-cancer deaths was observed.

Among the studies reporting conflicting results [37], [38]; *Reshko et al*. [29] conducted a similar dosimetric analysis of the heart in 75 early stage NSCLC and SCLC patients after SBRT and could not detect any dosimetric parameters of cardiac sub-structures associated with survival or non-cancer deaths, but showed that pre-existing cardiac disease was associated with increased number of cardiac events. A possible explanation for these contradicting results could be in addition to the relatively small patient number, pre-existing cardiac comorbidities, systemic cardiotoxic therapy and different techniques of cardiac sub-structure delineation. Standardized automated segmentation, as used in the present study, might reduce variability in segmentation and thereby lead to a better comparability. Furthermore, the time of onset and the pathophysiology of radiation-induced heart disease (RIHD), which involves changes of myocardial tissue and infiltration of immune cells after RT, remains poorly understood [39], [40], [41].

Some limitations apply to the present study, which are mainly associated with its retrospective character. As the present study has a relatively small sample size of 60 patients of a heterogeneous population with few events, conclusions must be drawn cautiously, especially concerning the causality between dose to cardiac sub-structures and non-cancer deaths and cardiovascular events. Detailed analysis of specific functional impact of dose delivered to the cardiac sub-structures may be limited, as ECGs, echocardiograms and assessment of coronary perfusion were not conducted routinely during follow-up. As such examinations are not part of clinical routine, clinical studies in ULCT may consider including additional cardiac examination before and after SBRT routinely. Furthermore, systemic cardiotoxic therapies in patient history and cardiac comorbidities at baseline might complicate to determine cardiac-specific mortality. Additionally, some limitations also apply to the dosimetric analysis of cardiac sub-structures and might explain why other relevant structures associated with non-cancer death in the literature, such as the left atrium or left ventricle, were not associated with CVE or non-cancer deaths. Furthermore, the accuracy of sub-structure delineation, especially for very small areas such as D_0.1cc_, has a large impact on the observed dose. Small discrepancies might lead to large observed dose differences, as in SBRT relatively small areas of the heart receive high doses of radiation. The anatomical proximity and the resulting dosimetric cross-correlation of the cardiac sub-structures make identification of relevant areas of the heart and the direct assessment of their impact on the clinical events problematic. Last but not least, the existence of competing risks should not be forgotten, as the patients in the present cohort had a limited survival in general, which could obscure potential long-time effects of dose to some of the sub-structures.

However, the present study included a rigorous follow-up including imaging every three months and frequent cardiological examination, if patients reported symptoms. Furthermore, the utilization of standardized deep-learning based auto-segmentation of the whole heart and cardiac sub-segments provides higher validity of the analyses and an excellent opportunity for improved comparability.

In conclusion, the present study cautiously indicates a possible association between dose delivered to the pulmonary artery and superior vena cava in the upper heart region and non-cancer-related death. The risk for RIHD and non-cancer deaths may be decreased by dose reduction and sparing to the aforementioned cardiac sub-structures. Further studies are required to determine dose limits for SBRT in UCLT, which is already associated with high rates of relevant toxicity to reduce the probability of cardiotoxicity.

Funding: Maiwand Ahmadsei and Sebastian M. Christ received support through the “Young Talents in Clinical Research” Beginner’s Grant from the Swiss Academy of Medical Sciences (SAMW) and the Bangerter-Rhyner Foundation.

## Declaration of Competing Interest

The authors declare that they have no known competing financial interests or personal relationships that could have appeared to influence the work reported in this paper.

## References

[1] Sung H., Ferlay J., Siegel R.L. (2021). Global cancer statistics 2020: GLOBOCAN estimates of incidence and mortality worldwide for 36 cancers in 185 countries. CA cancer J Clin.

[2] Guckenberger M., Andratschke N., Dieckmann K. (2017). ESTRO ACROP consensus guideline on implementation and practice of stereotactic body radiotherapy for peripherally located early stage non-small cell lung cancer. Radiother oncol J Eur Soc Ther Radiol Oncol.

[3] Chang J.Y., Mehran R.J., Feng L. (2021). Stereotactic ablative radiotherapy for operable stage I non-small-cell lung cancer (revised STARS): long-term results of a single-arm, prospective trial with prespecified comparison to surgery. The Lancet Oncology.

[4] Khorfan R., Kruser T.J., Coughlin J.M., Bharat A., Bilimoria K.Y., Odell D.D. (2020). Survival of primary stereotactic body radiation therapy compared with surgery for operable stage I/II non-small cell lung cancer. Ann Thorac Surg.

[5] Klement R.J., Hoerner-Rieber J., Adebahr S. (2018). Stereotactic body radiotherapy (SBRT) for multiple pulmonary Oligometastases: Analysis of number and timing of repeat SBRT as impact factors on treatment safety and efficacy. Radiother oncol J Eur Soc Ther Radiol Oncol.

[6] Palma D.A., Louie A.V., Rodrigues G.B. (2015). New strategies in stereotactic radiotherapy for oligometastases. Clin Cancer Res Off J Am Assoc Cancer Res.

[7] Palma D.A., Olson R., Harrow S. (2019). Stereotactic ablative radiotherapy versus standard of care palliative treatment in patients with oligometastatic cancers (SABR-COMET): a randomised, phase 2, open-label trial. Lancet Lond Engl.

[8] Palma D., Daly M., Urbanic J., Giuliani M. (2019). Stereotactic radiation for Ultra-Central lung tumors: Good idea, or Ultra-Risky?. Int J Radiat Oncol Biol Phys.

[9] Stam B., Kwint M., Guckenberger M. (2019). Subgroup survival analysis in stage I-II NSCLC patients with a central tumor partly treated with Risk-Adapted SBRT. Int J Radiat Oncol.

[10] Timmerman R., McGarry R., Yiannoutsos C. (2006). Excessive toxicity when treating central tumors in a phase II study of stereotactic body radiation therapy for medically inoperable early-stage lung cancer. J Clin Oncol Off J Am Soc Clin Oncol.

[11] Ahmadsei M., Christ S.M., Seiler A. (2022). Quality-of-life and toxicity in cancer patients treated with multiple courses of radiation therapy. Clin Transl Radiat Oncol.

[12] Christ S.M., Ahmadsei M., Wilke L. (2021). Long-term cancer survivors treated with multiple courses of repeat radiation therapy. Radiation Oncology.

[13] Darby S.C., Ewertz M., McGale P. (2013). Risk of ischemic heart disease in women after radiotherapy for breast cancer. N Engl J Med.

[14] Zamorano J.L., Lancellotti P., Rodriguez Muñoz D. (2017). 2016 ESC position paper on cancer treatments and cardiovascular toxicity developed under the auspices of the ESC committee for practice guidelines: the task force for cancer treatments and cardiovascular toxicity of the european society of cardiology (ESC). Eur J Heart Fail.

[15] Speirs C.K., DeWees T.A., Rehman S. (2017). Heart dose is an independent Dosimetric Predictor of Overall Survival in Locally Advanced Non-Small Cell Lung Cancer. J Thorac Oncol Off Publ Int Assoc Study Lung Cancer.

[16] McWilliam A., Kennedy J., Hodgson C., Vasquez Osorio E., Faivre-Finn C., van Herk M. (1990). Radiation dose to heart base linked with poorer survival in lung cancer patients. Eur J Cancer Oxf Engl.

[17] Stam B., Peulen H., Guckenberger M. (2017). Dose to heart substructures is associated with non-cancer death after SBRT in stage I-II NSCLC patients. Radiother Oncol J Eur Soc Ther Radiol Oncol.

[18] Bradley J.D., Paulus R., Komaki R. (2015). Standard-dose versus high-dose conformal radiotherapy with concurrent and consolidation carboplatin plus paclitaxel with or without cetuximab for patients with stage IIIA or IIIB non-small-cell lung cancer (RTOG 0617): a randomised, two-by-two factorial phase 3 study. The Lancet Oncology.

[19] McWilliam A., Abravan A., Banfill K., Faivre-Finn C., van Herk M. (2023). Demystifying the results of RTOG 0617: Identification of dose sensitive cardiac Subregions Associated With Overall Survival. J Thorac Oncol.

[20] Tohidinezhad F., Pennetta F., van Loon J., Dekker A., de Ruysscher D., Traverso A. (2022). Prediction models for treatment-induced cardiac toxicity in patients with non-small-cell lung cancer: A systematic review and meta-analysis. Clin Transl Radiat Oncol.

[21] Bergom C., Bradley J.A., Ng A.K. (2021). Past, present, and future of radiation-induced cardiotoxicity: refinements in targeting, surveillance, and risk stratification. JACC CardioOncology.

[22] Thor M., Deasy J.O., Hu C. (2020). Modeling the impact of cardiopulmonary irradiation on overall survival in NRG oncology trial RTOG 0617. Clin Cancer Res Off J Am Assoc Cancer Res.

[23] Zhang T.W., Snir J., Boldt R.G. (2019). Is the importance of heart dose overstated in the treatment of Non-Small cell lung cancer? A systematic review of the literature. Int J Radiat Oncol.

[24] Johnson-Hart C., Price G., Vasquez Osorio E., Faivre-Finn C., van Herk M. (2020). the impact of baseline shifts towards the heart after image guidance on survival in lung SABR patients. Radiother oncol.

[25] Chang E., Decker R.H., Hu X., Yu J.B., Gross C.P., Lester-Coll N.H. (2019). Predictors of toxicity from stereotactic body radiotherapy (SBRT) for lung tumors Ultra-Central or central to heart, esophagus, or proximal bronchial tree. Int J Radiat Oncol Biol Phys.

[26] Finnegan RN, Chin V, Chlap P, et al. Open-source, fully-automated hybrid cardiac substructure segmentation: development and optimisation. *Phys Eng Sci Med*. Published online February 13, 2023. 10.1007/s13246-023-01231-w. 10.1007/s13246-023-01231-w PMC10030448 36780065

[27] Louie A.V., van Werkhoven E., Chen H. (2015). Patient reported outcomes following stereotactic ablative radiotherapy or surgery for stage IA non-small-cell lung cancer: Results from the ROSEL multicenter randomized trial. Radiother Oncol J Eur Soc Ther Radiol Oncol.

[28] Isensee F., Jaeger P.F., Kohl S.A.A., Petersen J., Maier-Hein K.H. (2021). nnU-Net: a self-configuring method for deep learning-based biomedical image segmentation. Nature Methods.

[29] Reshko LB, Kalman NS, Hugo GD, Weiss E. Cardiac radiation dose distribution, cardiac events and mortality in early-stage lung cancer treated with stereotactic body radiation therapy (SBRT). *J Thorac Dis*. 2018;10(4). 10.21037/jtd.2018.04.42. 10.21037/jtd.2018.04.42 PMC5949491 29850140

[30] Farrugia M., Yu H., Ma S.J. (2022). Right atrial dose is associated with worse outcome in patients undergoing definitive stereotactic body radiation therapy for central lung tumors. Cancers.

[31] Wong O.Y., Yau V., Kang J. (2018). Survival impact of cardiac dose following lung stereotactic body radiotherapy. Clin Lung Cancer.

[32] Ma J.T., Sun L., Sun X. (2017). Is pulmonary artery a dose-limiting organ at risk in non-small cell lung cancer patients treated with definitive radiotherapy?. Radiation Oncology.

[33] Han C.B., Wang W.L., Quint L. (2014). Pulmonary artery invasion, High-Dose radiation, and overall survival in patients With Non-Small Cell Lung Cancer. Int J Radiat Oncol.

[34] Nagayasu T. (2010). Importance of follow-up inspection after pulmonary angioplastic procedures for lung cancer surgery. General Thoracic and Cardiovascular Surgery.

[35] Yamashita M., Komori E., Sawada S. (2010). Pulmonary angioplastic procedure for lung cancer surgery. General Thoracic and Cardiovascular Surgery.

[36] Rodriguez Pla M., Dualde Beltrán D., Aliaga Chueca A. (2022). PO-1239 heart substructures delineation in central lung SBRT and cardiac toxicity. Radiotherapy and Oncology.

[37] Rodríguez Plá M., Dualde Beltrán D., Aliaga Chueca A. (2021). Heart substructures delineation in lung SBRT: Central and Ultra-Central lesions. Biomed J Sci Tech Res.

[38] Kearney M., Keys M., Faivre-Finn C., Wang Z., Aznar M.C., Duane F. (2022). Exposure of the heart in lung cancer radiation therapy: A systematic review of heart doses published during 2013 to 2020. Radiother Oncol.

[39] Cahlon O., Khan A.J. (2017). Cardiac toxicity: the more we learn, the less we know. Int J Radiat Oncol Biol Phys.

[40] Schultz-Hector S., Trott K.R. (2007). Radiation-induced cardiovascular diseases: is the epidemiologic evidence compatible with the Radiobiologic data?. Int J Radiat Oncol Biol Phys.

[41] Wu W., Masri A., Popovic Z.B. (2013). Long-term survival of patients with radiation heart disease undergoing cardiac surgery: a cohort study. Circulation.

